# Deviations from the inverse relationship between tumor and normal tissue dose and RBE in fast neutron data are compatible with the presence of hypoxia, with possible applications to hypo-fractionated proton therapy

**DOI:** 10.3389/fonc.2026.1792868

**Published:** 2026-05-07

**Authors:** Bleddyn Jones

**Affiliations:** 1 Gray Laboratory, Oxford Institute for Radiation Oncology and Biology, Oxford, United Kingdom; 2 Green Templeton College, Oxford, United Kingdom

**Keywords:** hypo-fractionated proton therapy, radiation radiobiology, RBE-Dose relationship, relative biological effectiveness, tumor hypoxia compartments

## Abstract

**Aim of study:**

Fast neutron data from Hammersmith (UK) showed that the inverse relationship between dose and RBE (Relative Biological Effectiveness) is not maintained in many *in vivo* situations, originally attributed to hypoxic effects. The present study aims to simulate these RBE patterns in two cancers and one normal tissue for neutrons as well as protons.

**Methods:**

A new model uses LQ formulism with radiosensitivity changes increasing with LET and with LET-related reductions in hypoxia. Inequalities are defined for the parameter ratios used to estimate RBEs in oxic and hypoxic compartments, with the overall RBE being also determined by the initial hypoxic fraction and the compartmental surviving fractions with increasing dose. Extrapolation from neutrons to protons is assumed by using a 90% RBE reduction for the excess of RBE beyond unity. Also, the separate influence of the dose rate effect on RBE at higher doses is investigated.

**Results:**

For normal skin, the C3H mouse mammary carcinoma and the Ehrlich ascites cancer, three quite different RBE responses are fitted by the model within practical statistical bounds. The overall RBE initially follows the oxic curve but shifts with increasing dose to the higher hypoxic RBE curve, the response depending mostly on the pretreatment hypoxic fraction and other hypoxia parameters. Such patterns of RBE changes are also obtained for protons, but cannot be obtained from the dose rate effect at higher dose, although the DNA repair rate will increase RBE with dose.

**Conclusions:**

This tentative but complex radiobiological modelling study shows that the Hammersmith neutron RBE results can be simulated by the presence of two physiological compartments and that similar RBE changes may occur for protons. Further experimental research is suggested for determining if protons do show effects at similar dose ranges, which could be relevant for hypo-fractionated or single fraction schedules.

## 1 Introduction

Clinical interest in proton-based radiotherapy continues to increase, due to its more selective dose distributions associated with the Bragg-peak effect, although many forms of photon-based radiotherapy are achieving more conformal dose distributions as well as enabling treatment to be given in fewer fractions, the extreme example being ‘radiosurgery’ given in a single highly focused treatment utilizing rotating arcs. If proton therapy is to compete in cost effective terms, the use of hypo-fractionation probably will be necessary.

The relative biological effectiveness (RBE) of protons and photons differ, such that lower physical doses of protons are required to provide the same biological effect depending on the operative RBE. A wealth of *in vivo* RBE-related research was previously done using fast neutron therapy, particularly in the UK and from which important insights can be found (1). Dr Hal Gray thought that neutrons were an important tool for radiobiology research rather than therapy (OCA Scott, personal communication). The Hammersmith Medical Research Council cyclotron experiments provided RBE values of usually between 2–3 in many normal tissue experiments (2), and in experimental tumors as summarized by Field (3). Similar values were found in *in vivo* cellular based assays as by Hendry, Major and Greene (1975). Fast neutrons cause tissue ionization mostly by formation of recoil protons, the energy spectrum of which is similar to the proton therapy energies found near to the Bragg peak region (Jones, 2001), such that proton RBE models can be used to predict neutron RBE values.

The above experiments showed a marked inverse relationship between fast neutron dose per fraction and RBE, the latter being amenable to modelling by modification of the linear quadratic model (4). However, two potentially important exceptions were found. In normal skin experiments, but not in the other tissues studied, it was possible to increase the single fraction neutron dose to around 17 Gy, revealing a probable minimum value of RBE at around 10–12 Gy with further substantial increments in RBE beyond that dose (2). Also, the many experimental tumor studies summarized by Field (3) showed an initial reduction in RBE with dose towards minimum RBE values at around 6–8 Gy, followed by increasing RBE at higher doses. These publications are not widely available but are reprinted in two books (5, 6), Since the applied fractional doses used the many fast neutron trials were between 0.9-1.6 Gy [see 1] and were much lower than these local RBE nadir regions, it was not necessary to utilize this information in practice. The observed patterns of RBE changes were attributed to the presence of hypoxia (6), but no simulations have been published.

However, it is possible that such nadir RBE values could occur in the context of proton therapy given in hypo-fractionated forms. The analysis of the neutron studies with extrapolation to the lower RBE values found with protons is attempted in this report by means of assuming two subpopulations of cells: the well oxygenated compartment with lower RBE values, and a hypoxic compartment with inevitable higher RBE values. The overall RBE will be determined by the percentage of hypoxic cells, i.e. the hypoxic fraction, which will increase with dose per fraction due to the higher radiation cell killing of oxic cells by lower doses and with increasing dose a shift between the oxic RBE to that of the higher hypoxic RBE.

It is necessary to understand some basic principles of radiobiology concerning neutrons and the oxygen effect. Neutron and proton RBE is increased in proportion to the local clustering (or density) of ionization, resulting in increased complexity of DNA molecular disruption (strand breakage, and base damage which can lead to lethal chromosomal breaks), such that DNA repair enzymes can be progressively rendered less effective so that radiosensitivity increases. Also, the repair mechanisms are known to shift with increasing LET to more complex systems, from non-homologous end-joining to that of recombination repair, but such changes will be inherently included in the assumed radiosensitivity parameter changes. The standard quantification of ionization clustering along radiation tracks is expressed as the linear energy transfer (LET). The LET for protons is generally lower than for a fast neutron beam but the two may be similar in the Bragg peak region.

Essentially, oxygen acts as an amplifier of low ionization density radiations due to enhanced free radical production in cellular water, causing enhanced radiosensitivity. Consequently, the oxygen effect on radiosensitivity is reduced with radiations where local ionization density is higher, causing oxygen-independent enhanced radiosensitivity. Thus, the so called ‘oxygen effect’ on radiosensitivity modification can change the iso-effective dose requirement by as much as a factor of 2–3 with clinically used megavoltage photons, but this factor can be reduced to around 1.4 to 1.8 with neutrons *in vitro*, but with more minimal reductions in the case of spread-out Bragg-peak protons. The degree of reduction will be more variable in living tissues and tumours due to the heterogeneity present with complex oxygen gradients when compared with *in vitro* experiments using different nuclear particles and ions by various authors (Barendsen et al. (7), Berry (8), Raju and Jett (9) and Skarsgard (10). The extent to which the oxygen effect is reduced depends on the LET, the effect diminishing steeply if exceeding 10 keV.μm^-1^, so that protons are expected to have minimal deviations from megavoltage photons, unlike with neutrons. The tabulated data of Furusawa et al. (11) on the measured α radiosensitivity parameter in oxic and hypoxic conditions using different ion beams with increasing LET can be seen graphically in Jones (12).

A further possible explanation for increasing RBE with increasing dose could be caused by the dose rate effect, where biological effects can diminish by prolongation of the treatment exposure times relative to the half-times of the DNA repair enzymes, requiring increased doses to maintain an isoeffect with a resulting increase in RBE. This possibility is tentatively explored below.

## 2 Methods and description of models

### 2.1 Higher LET particle therapy radiations

In neutron and proton radiobiology, radiosensitivities increase with LET, causing RBE to increase. It is known, mainly from neutron radiobiology that fast neutrons, when compared to photon megavoltage radiation, both α and β parameters increase, but the former to a greater extent than the latter (1). For many years it was accepted that the β parameter did not change, due to experiments being performed in only one cell line where deviation was minimal (13). It is reasonable to assume that protons will behave similarly since fast neutron beam ionisations are mostly due to the formation of recoil protons from hydrogen nuclei in cellular water and other hydrogen containing molecules. Indeed, the ionization energy released (KERMA, or kinetic energy released per unit mass) and dose is proportional to the ‘hydrogen density’ of biological and other materials (see 1).

Comparison of neutron or proton dose to the reference photon dose (BED) per fraction is best achieved by using Equation 1, which expresses isoeffective cell killing by two radiations of different LET values, and where the same number of fractions are given by photons and protons:

(1) $$\alpha_{L}d_{L}+\beta_{L}d_{L}^{2}=\alpha_{H}d_{H}+\beta_{H}d_{H}^{2}$$

The subscripts L and H refer to low and higher LET conditions respectively for the radiosensitivity parameters α (the coefficient of cell killing per unit dose) and β (the coefficient of cell killing per unit dose squared), where the reference radiation (usually photons) is of lower LET. Since the killing response is dominated by α parameters at near zero dose and by the β parameters at very high doses, consequently the RBE (which is the ratio of $\frac{d_{L}}{d_{H}}$ for a specified isoeffect) will approximate at very low dose to a maximum value approaching the ratio $\frac{\alpha_{H}}{\alpha_{L}}$ (the RBEmax) and at very high doses to the ratio $\frac{\sqrt{\beta_{H}}}{\sqrt{\beta_{L}}}\;$(the RBEmin).

The inclusion of the oxygen enhancement ratio (OER) is achieved by dividing the radiosensitivity parameters α and β by hypoxic reduction factors q_α_ and q_β_ to respectively divide the cell killing parameters The linear quadratic cell killing effectiveness is then reduced as in:

(2) $$\frac{\alpha }{q_{\alpha }}d+\;\frac{\beta }{q_{\beta }}d^{2}$$

It is known that the oxygen effect (OER) is larger for the β parameter than for the α parameters, so that q_β_ exceeds q_α_, as used by Nahum et al. (14), and which effectively allows for the OER to be greater at higher doses per fraction than at lower doses as found experimentally by (Skarsgard & Harrison (15), in the model.

The cell kill in hypoxic states for the higher LET radiations (neutrons and protons) can then be modified by using different values of q denoted by a further subscript H, such that in higher LET conditions the q values are inevitably reduced since the oxygen effect is progressively diminished. The values of q_αH_ and q_βH_ will be smaller for neutrons than those for protons, and q_αH_ will be less than q_βH_, just as q_α_< q_β_.

The approximated RBE limits in hypoxia are then modified to be.

(3) RBEmax=αHqαHαLqα

(4) RBEmin=βHqβHβLqβ

which respectively indicate that RBEmax is increased by a factor of $\frac{q_{\alpha }}{q_{\alpha H}}$ and RBEmin by a factor of $\sqrt{\frac{q_{\beta }}{q_{\beta H}}}$.

### 2.2 Dose rate effects

The modelling of dose rate effects in the low dose rate range (for example 0.5–25 Gy hr^-1^) is achieved by applying the sublethal damage continuous repair equation to only the β parameter terms as.

(5) $$\alpha_{L}d_{L}+\beta_{L\;}d_{L}^{2}\;f(\mu t)=\alpha_{H}d_{H}+\beta_{H}\;d_{H\;}^{2}f(\mu t)$$

Where *t* is the treatment duration and *f(μt)* is a complex exponential function containing an averaged repair rate constant μ which is related to the half time of repair as $\frac{0.693}{T_{half}}$ (16).

The much longer times taken to deliver the reference radiation will require an increase in the dose for a specified isoeffect and so the numerator of the RBE expression ( $RBE=\frac{Dose_{L}}{Dose_{H}}$), will increase causing the relationship between RBE with dose per fraction not to reduce by as much as expected at higher doses, especially because the change in total dose required with the higher LET radiation will be less due to its higher dependency on the dose-rate insensitive α parameter.

### 2.3 Experimental data sets where RBE does not follow a purely inverse relationship with dose

The experimental data is obtained from Field and Hornsey and coworkers at the Medical Research Cyclotron Unit at the Hammersmith Hospital, as summarized by 3 and Hornsey (2); they are reproduced by Andrews (5). Their fast neutron RBE studies showed that the RBE reduces with increasing dose but can increase at higher doses e.g. in skin for neutron doses above 15 Gy. Other normal tissue were not irradiated to the same high doses. This finding could be due either to the small hypoxic fraction in normal skin manifesting as a higher RBE at high dose, or due to a dose rate effect which also manifests at higher dose (due to longer treatment exposure times), and these alternatives are amenable to modelling. Another similar phenomenon occurs with some experimental tumors where RBE reduces with neutron dose per fraction but at doses of around 8 Gy the RBE increases. This is also could be because lower doses will preferentially eliminate oxic cells whilst hypoxic cells remain to be killed at the higher doses, and are more efficiently eliminated by neutrons than photons, causing the effective RBE to increase since hypoxic cell populations will have a higher RBE than oxic populations, as shown further below.

For the skin tissue data, neutron doses and RBE values can be used directly from the published Hammersmith data set, but for the experimental tumor data, points were obtained from the published continuous curves (which will inevitably underestimate the degree of statistical variation in the original data), but this is a reasonable approach for exploratory modelling purposes. To introduce statistical variation, 95% confidence limits were used around the estimated mean RBE predictions.

The three representative examples chosen, namely skin, the C3H mammary carcinoma and the Ehrlich ascites (another mammary carcinoma) provide a wide range of pre-treatment hypoxia values, respectively from low, intermediate to high values. These are next considered in further detail.

Skin is known to contain hypoxic regions as confirmed by the pioneering electrode studies of Cater and Silver (17) and confirmed by other techniques in later studies (18). The layers of skin vary in their oxygenation, the superficial layer and the appendages being lowest, and all can be influenced by ambient temperature, hence why skin becomes blue (due to reduced haemoglobin) and eventually white with increasing cold. For laboratory conditions a radiologically significant hypoxic fraction of around 1.5% is assumed.

The C3H mammary carcinoma develops an increasing hypoxic fraction with tumor volume growth, due to increasing diffusion related hypoxia, enlarging necrotic regions, with associated tumor vascular thrombosis, haemorrhage and intermittent vascular closure. It can be between 7 and 30%, as outlined in some studies where, for example a 200 mm*^3^* tumor contains 23% hypoxia (19, 20). It is not known what the dimensions of the Hammersmith tumors were. The experiments were done many years earlier before extended passages of transplanted tumors are likely to select more rapidly growing cells with earlier onset of hypoxia.

The Ehrlich ascites tumor is derived from a mammary carcinoma but grows mostly in the fluid of the peritoneal cavity away from a direct vascular system, so that diffusion related hypoxia predominates. It is reported to have a high hypoxic fraction and a median oxygen tension of around 1mm Hg which would cause radiological hypoxia (21). This suggests that more than 50% of the cells will be hypoxic, but some cells adherent to the peritoneal surface can acquire a vascular input, so that experiments may be influenced by a slightly lower hypoxic fraction. Ehrlich ascites is also known to have a higher RBE (due to hypoxia) than in normal tissues (22).

The historical neutron data shows that the theoretical expectation of a reduced RBE with increasing dose per fraction is not upheld in all biological systems: tumors with a high hypoxic fraction probably show the largest deviation, but so can normal skin tissue at very high dose per fraction and where the hypoxic fraction in standard laboratory conditions is probably very low (reactive skin vasoconstriction with resulting hypoxia occurs in cold conditions, e.g. causing human skin to become blue or white).

Radiosensitivity values are probably lower for *in vivo* irradiations, since *in vitro* growing conditions are optimized in terms of gaseous and nutrient supplies. The estimated values for human normal tissue, for example were extremely low (23). In the present study the values of α are consequently low, although this makes no difference to the RBE which is dependent on the ratio of the α parameter at high and low LET.

### 2.4 Description of model

A new radiobiological model has been constructed to include the essential operative parameters: it allows for a higher RBEmax and RBEmin in hypoxic cells compared to those in oxic cells, due to the influence of a reduced OER with a high LET radiation. The final part of the computation allows for an increasing hypoxic fraction with dose, due to the more effective elimination of oxic cells at lower doses.

The computing steps are numerous and described as follows:

The surviving fractions for oxic cells after lower LET exposures are:

(6) $$SF_{xox}=e^{-(\alpha_{L}\;d_{L}-\beta_{L}d_{L}^{2})}$$

The surviving fractions for oxic cells after higher LET exposures are.

(7) $$SF_{nox}=e^{-(\alpha_{H}\;d_{H}-\beta_{H}d_{H}^{2})}$$

The RBE in oxic conditions (RBE_ox_) is found from the positive root solution for *d_L_* in.

(8) $$\alpha_{L}\;d_{L}+\beta_{L}d_{L}^{2}=\alpha_{H}\;d_{H}+\beta_{H}d_{H}^{2}$$

and dividing by the variable *d_H_* (see Appendix).

Similarly, the surviving fractions for hypoxic cells after the reference radiation (lower LET) exposures are:

(9) SFxhyp=e−(αL' dL−βL'dL2)

Where αL'=αLqα and βL'=βLqβ, (q_α_ and q_β_ are the hypoxia reduction factors for the low LET α and β respectively).

The surviving fractions for hypoxic cells after higher LET neutron exposures are.

(10) SFnhyp=e−(αH' dH−βH'dH2)

And where, similarly, αH'=αHqαH and βH'=βHqβH, (where q_αH,_ and q_βH_ are the modified hypoxia reduction factors for the higher LET α and β parameters respectively due to diminution of the OER with increasing LET).

If *h* is the hypoxic fraction of cells, then the combined surviving fraction in oxic conditions will be.

(11) $$CSF_{ox}=(1-h)\;SF_{ox}+(1-h)\;SF_{nox}$$

and the combined surviving fraction in hypoxia:

(12) $$CSF_{hypox}=h\;SF_{xhyp}+h\;SF_{nhyp}$$

The RBE in hypoxia (RBE_hypox_) is obtained by taking the positive root of the solution for *d_L_* in.

(13) αL' dL−βL'dL2=αH' dH−βH'dH2

(as given in the Appendix), and then dividing by the variable *d_H_*.

The combined surviving fraction in hypoxic conditions will be.

(14) $$CSF_{ox}=x\;SF_{ox}+x\;SF_{nox}$$

The fraction of hypoxic cells (*f_hyp_*) will vary with increasing dose as the ratio of.


$$\frac{CSF_{hyp}}{\left(CSF_{ox}+CSF_{hyp}\right)}$$

and the fraction of oxic cells (*f_ox_*) is expressed by.


$$\frac{CSF_{ox}}{\left(CSF_{ox}+CSF_{hyp}\right)}$$

The effective (or combined) RBE for the entire population of cells is then given by.

(15) $$RBE_{eff}=f_{ox}\;RBE_{ox}+f_{hyp}\;RBE_{hyp}$$

It is instructive to consider the *RBE_ox_, RBE_hypox_* and *RBE_eff_* outputs on the same graphic in order to observe their relationships. The above equations are all linked to allow graphical outputs within Mathematica (Wolfram, USA) software. The resultant expressions are long and contain multiple instances of each of the 9 radiobiological parameters and the operative dose. Copies of the programme are available from the present author on request.

Radiobiological constraints are required, which ensure an inverse relationship of RBE with dose for each physiological compartment, as follows:

(16) αLβL(ox)<αLβL(hypox)<αHβH(ox)<αHβH(hypox)

The limits of RBE at near zero and very high doses are given in **Table 1**. These represent the asymptotes on the ordinate and abscissa of the RBE graphical estimations for the oxic and hypoxic RBE curves and are useful in guiding the initial choice of parameters within the mandatory radiobiological constraints given in Equation 16.

**Table 1:** Limits of RBE in oxic and hypoxic conditions at low and high dose.

	Oxic	Hypoxic
Low Dose Asymptote (or near zero dose)	$\frac{\alpha_{H}}{\alpha_{L}}$	$\frac{\alpha_{H}q_{\alpha L}}{\alpha_{L}q_{\beta H}}$
High Dose Asymptote (as dose becomes large)	$\sqrt{\frac{\beta_{H}}{\beta_{L}}}$	$\sqrt{\frac{\beta_{H}q_{\beta L}}{\beta_{L}q_{\beta H}}}$

It should be noted here that it is the ratios of the parameters between the high and low LET and between the oxic and hypoxic cells will govern the responses.

The data sets are then fitted with realistic radiosensitivity and oxygen-related parameters that comply with the above constraints, and considering the fact that the Hammersmith neutron energy of 16 MeV was lower and therefore would be associated with high RBE values compared to those using a 64 MeV beam (24), and in the order of α_L_, β_L_, α_H_, β_H_, whilst respecting the above inequalities, and using the same values of q_α_=1.75 and q_β_ =3.25 for an adenocarcinoma as in Nahum et al. (14). The extent to which oxygen changes radiosensitivity appears to be robust throughout the animal kingdom: OER values in murine and human cells are not known to differ substantially. Values of the q_αH_ and q_βH_ are entered finally to obtain the most reasonable fit by visual inspection: they will be reduced in comparison to the full oxygen enhancement ratio (as given by q_α_ and q_β_), being smaller to an extent proportional to their LET values, with the least change for protons (due to their relatively low LET) and the greatest change for neutrons (due to their higher LET). No attempt has been made to use further mathematical optimization or statistical fitting in this exploratory multi-parameter study because of the overall complexity of the final equations and the relative lack of data points compared to free parameters in the tumor examples, the intent being to find if reasonable parameters which can provide curves which closely resemble the original data sets. Changes in q_αH_ and q_βH_ cause y-axis scaling shifts to the curves.

### 2.5 Conversion of neutron to proton RBE values

The modelled proton irradiations are assumed to reflect the operative radiobiology at the just beyond the mid spread-out Bragg-peak such that much lower RBEs will be found than for fast neutrons but with potential for being higher than the conventional constant RBE of 1.1, as found experimentally and with publication of radiosensitivity parameters by Warenius & Britten (24) for neutrons, Britten et al. (25) and Calugaru et al. (26) for protons. Since proton energies can be used to model single-fraction neutron RBE data (12) by an effective scaling process due to the fact that fast neutrons cause most of their ionization by formation in cellular water of recoil protons with sufficient ranges to cause DNA damage, provides justification to apply a single conversion factor of 0.1 to the excess neutron RBE above 1, which then provides reasonable proton RBE values for tentative modelling purposes. Such a method will inherently include dose per fraction effects since they are included in the neutron RBE data but will inevitably result in the minimum RBE occurring at the same proton dose as for neutrons, which might as a *prime facie* case be objected to as a working hypothesis, although there are further reasons given below to accept that very similar results can be obtained with protons. It is emphasized that the absolute values of proton RBE after the scaling process are not critical in the present tentative modelling study, because it is the change in RBE with dose that is of primary interest and the occurrence of minimum RBE points at certain doses.

The assumed proton radiosensitivity and hypoxia reduction factors require modification, so that deviations from their reference (photon) counterparts are much smaller than in the case of neutrons since the proton LET is smaller than the neutron LET.

It is appreciated that ascites will not be treated by proton therapy but the experimental model provides interesting radiobiological insights.

### 2.6 Hypoxic fractions

The hypoxic fractions used in the study were obtained by the graphical fitting process and in iterative data fitting analysis were chosen to be within the expected ranges for each tissue or tumor studied, as given already above.

## 3 Results

The radiosensitivity parameters, α and β given below are in units of Gy^-1^ and Gy^-2^ respectively and are not reproduced further. The parameter q is dimensionless, and the hypoxic fraction (*h*) is given as a percentage in each instance below.

### 3.1 The three neutron data sets

**Figure 1:**
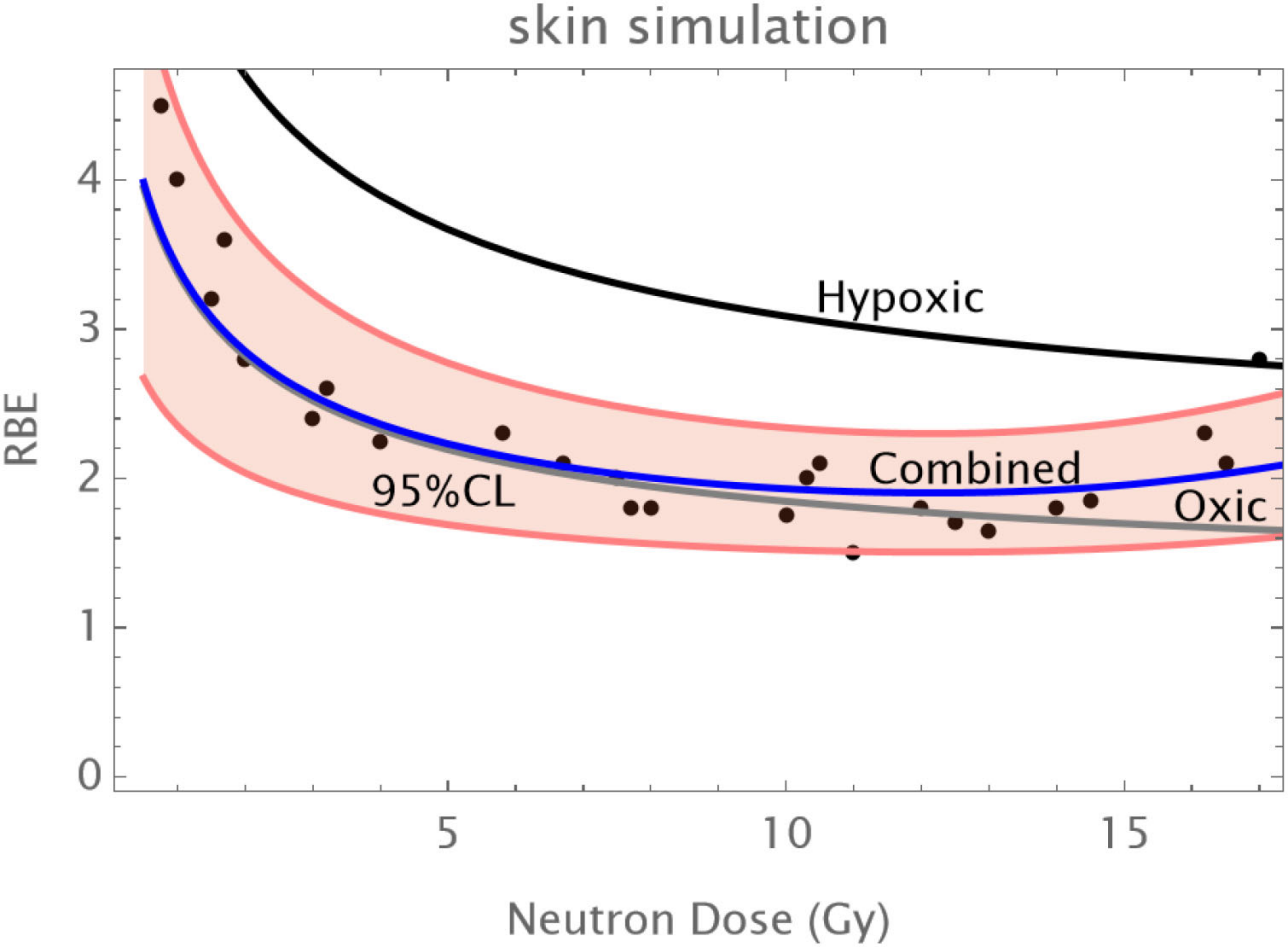
Data points at Hammersmith for normal skin treated by neutrons. Assumed parameters: α_L_=0.15; a_H_=082; β_L_=0.03; β_H_ =0.05; q_α_=1.75;q_β_=3.25; q_αH_=1.1; q_βH_=1.2; *h* = 1.5%. The hypoxic RBE curve is shown as black, the oxic RBE as grey and the combined RBE as blue. The pink shaded zone represent the assumed 95% confidence limits for a coefficient of variation of 22% which encompasses all but one point.

**Figure 2:**
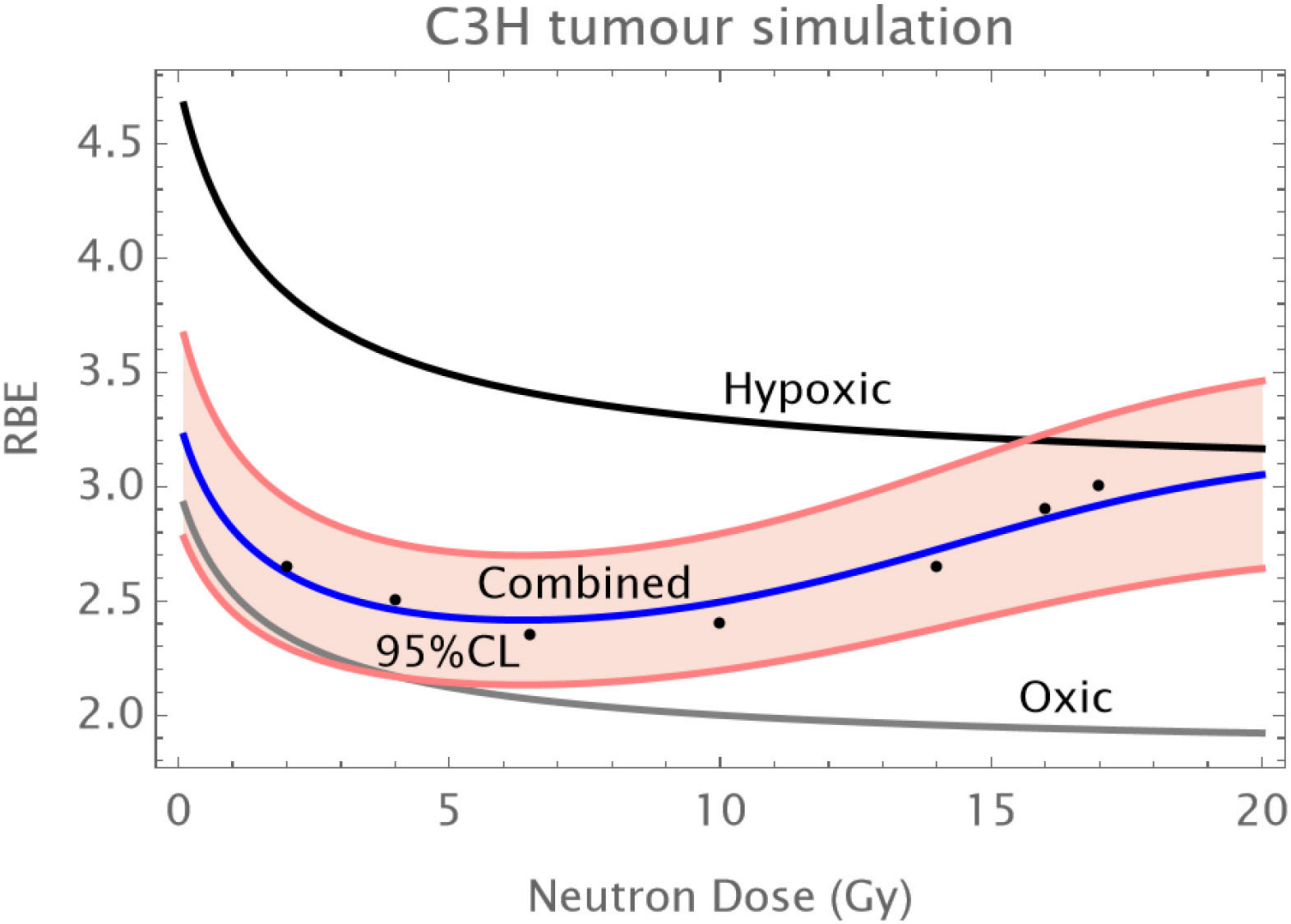
RBE changes in a mouse mammary cancer. Data points in black; the grey curve is that of oxic cells, the black curve of hypoxic cells, the overall RBE response is given by the blue curve. Here a larger hypoxic fraction of 10% is assumed with a coefficient of variation of 10%. The fitting is provided by α_L_=0.1; a_H_=0.32; β_L_=0.015; β_H_ =0.05; q_α_=1.75;q_β_=3.25; q_αH_=1.15; q_βH_=1.45; *h* = 0.1.

**Figure 3:**
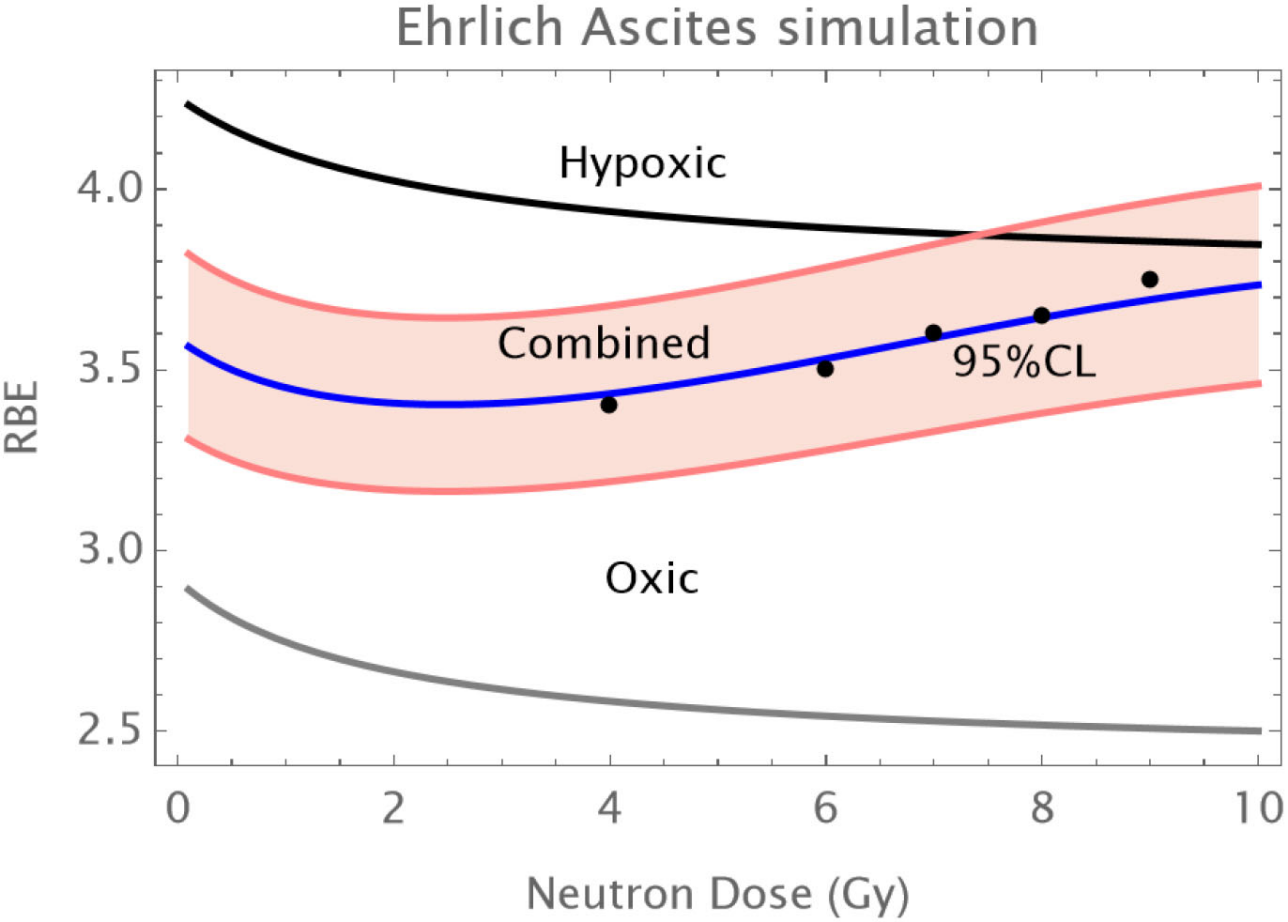
RBE changes in the Ehrlich ascites tumor at Hammersmith. The data is limited to between 4 and 9 Gy, but the models are extrapolated in order provide insight into the potential mechanism. Data points in black; the grey curve is that of oxic cells, the black curve of hypoxic cells, the overall RBE response is given by the blue curve. The assumed coefficient of variation of 5% is used to provide 95% confidence limits, the hypoxic fraction is 50%, with the fitting provided by α_L_=0.12; a_H_=0.35; β_L_=0.012; β_H_ =0.07; q_α_=1.75;q_β_=3.25; q_αH_=1.2; q_βH_=1.35; *h* = 0.5.

1. Normal Skin: An initial reduction in RBE with dose is followed by an upturn in RBE at doses of above 12–14 Gy as shown in **Figure 1**.
2. The C3H mouse mammary tumor is known to contain a significant proportion of hypoxic cells and develops necrotic cords. In this carcinoma a minimum RBE is seen at around 6 Gy of fast neutrons, followed by a progressive rise in RBE as shown in **Figure 2**.
3. The Ehrlich Ascites tumor is known to contain considerable hypoxia and here the limited dose range studied has an increasing RBE with dose. A hypoxic fraction of 50% is assumed in **Figure 3**. The modelling is extended to show a probable minimum RBE at a dose lower than used in the data set.

### 3.2 Proton simulations

**Figure 4:**
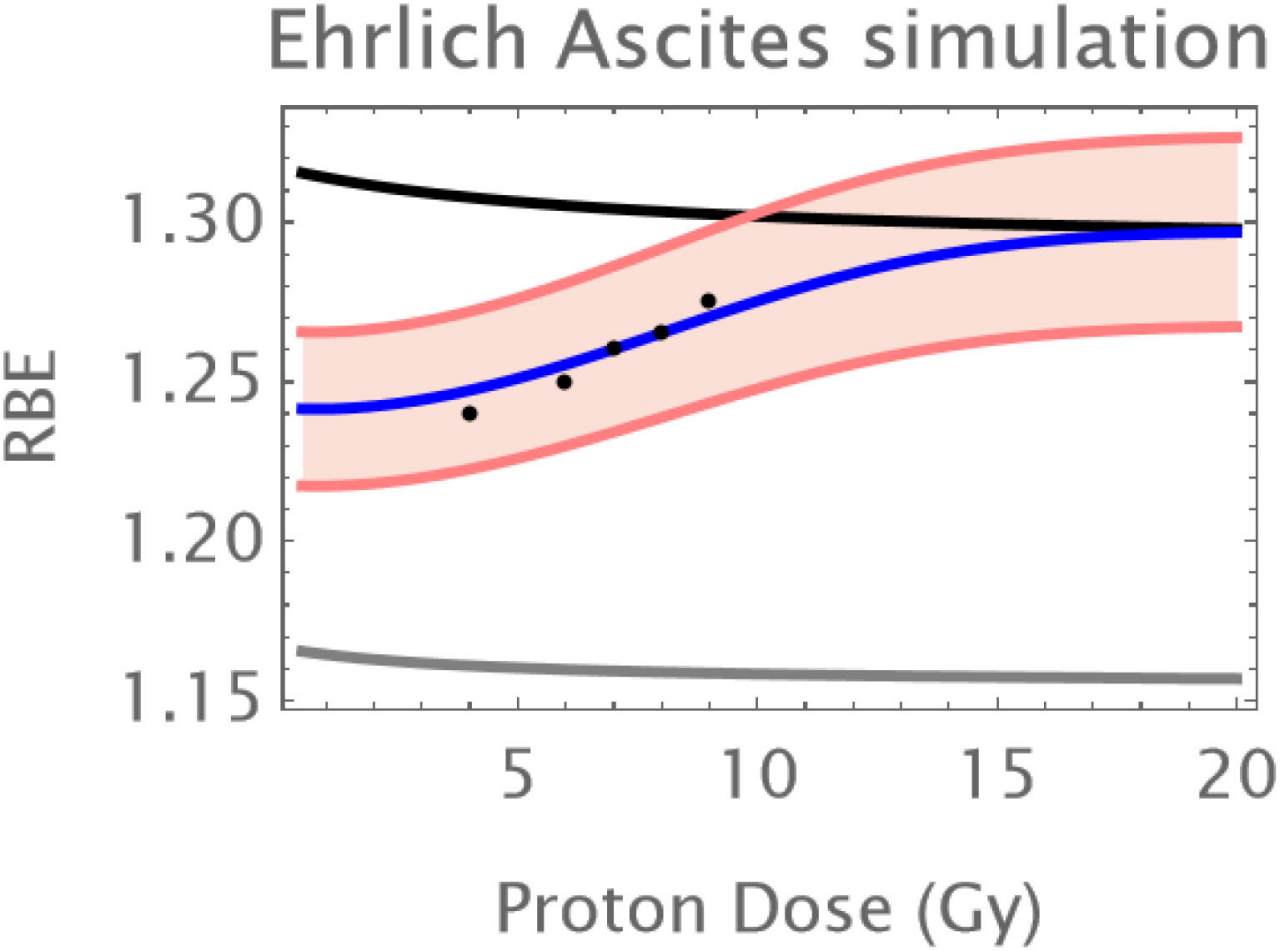
The Ehrlich ascites proton RBE simulation with parameters α_L_=0.12; a_H_=0.155; β_L_=0.012; β_H_ =0.016; q_α_=1.75;q_β_=3.25; q_αH_=1.55; q_βH_=2.6; *h* = 0.5. The coefficient of variation is the same as in **Figure 3**. Data points in black; the grey curve is that of oxic cells, the black curve of hypoxic cells, the overall RBE response is given by the blue curve.

**Figure 5:**
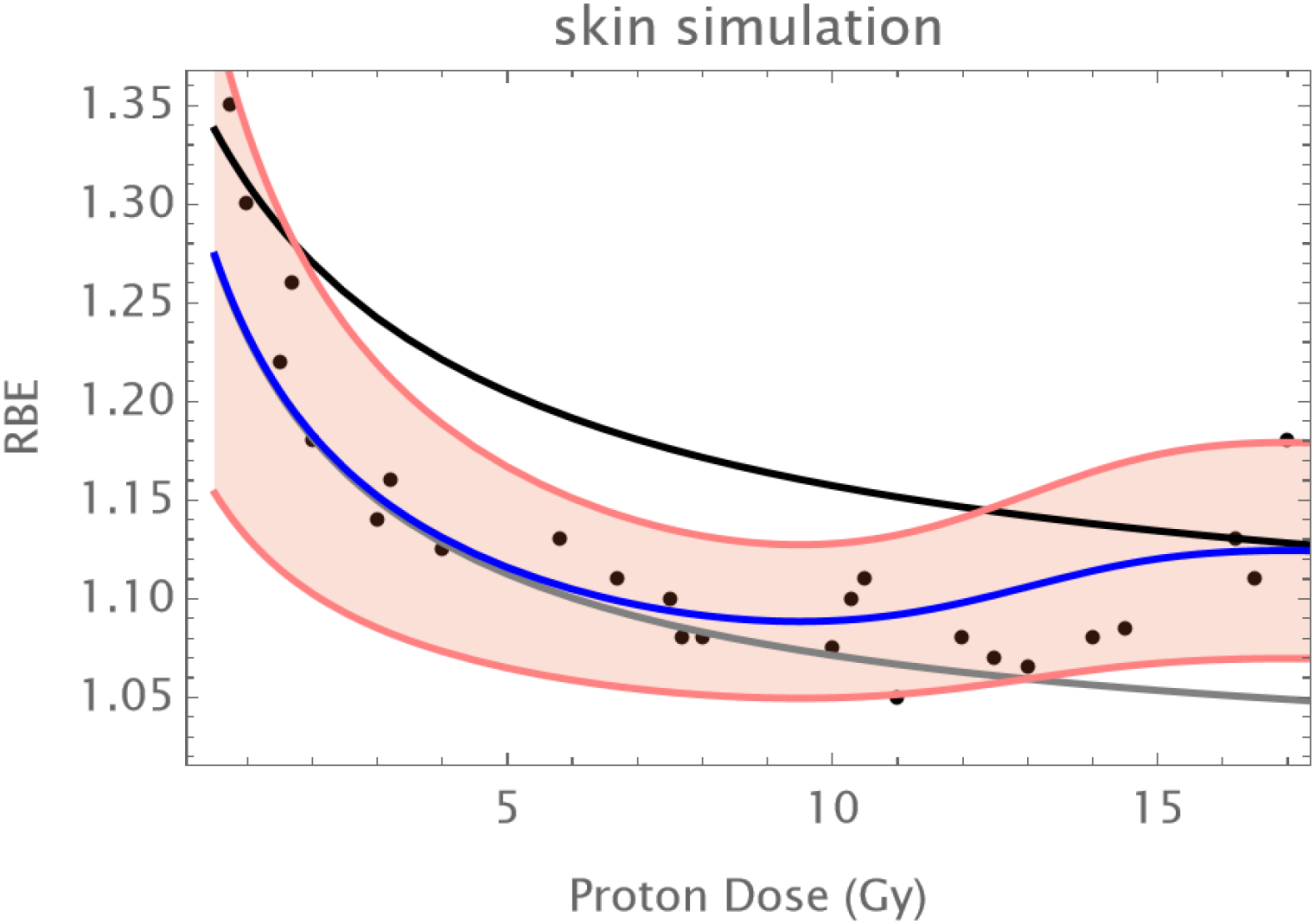
Simulated skin RBE changes with proton dose: data points of Field are modified to a typical proton range. α_L_=0.15; a_H_=0.2; β_L_=0.03; β_H_ =0.0305; q_α_=1.75;q_β_=3.25; q_αH_=1.7; q_βH_=2.9; *h* = 0.015. The coefficient of variation is the same as in **Figure 1**. Data points in black; the grey curve is that of oxic cells, the black curve of hypoxic cells, the overall RBE response is given by the blue curve.

**Figure 6:**
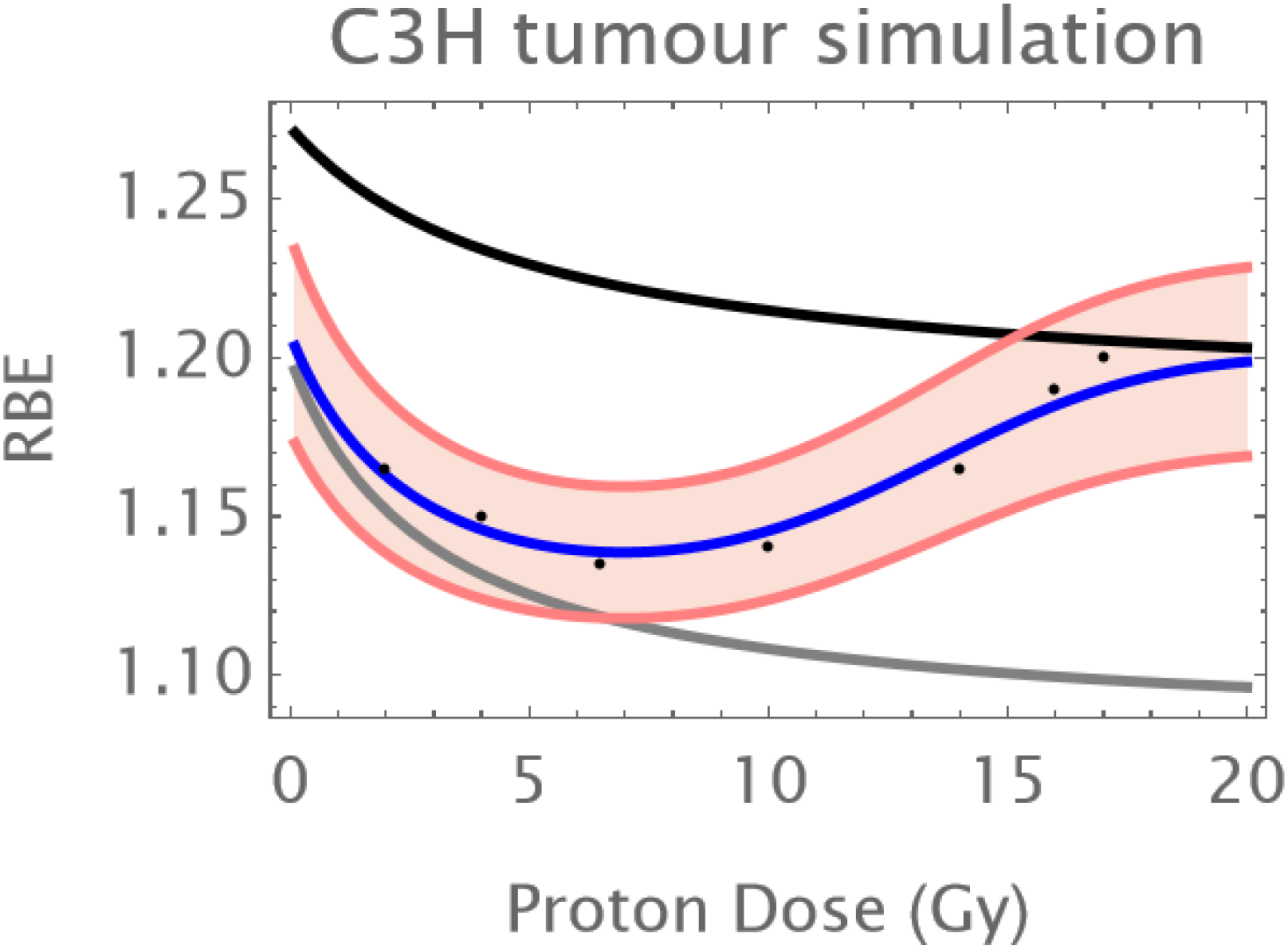
Simulated RBE changes with proton dose for C3H mammary carcinoma, with parameters α_L_=0.1; a_H_=0.12; β_L_=0.015; β_H_ =0.0175; q_α_=1.75;q_β_=3.25; q_αH_=1.65; q_βH_=2.7; *h* = 0.1. The coefficient of variation is the same as in **Figure 2**. Data points in black; the grey curve is that of oxic cells, the black curve of hypoxic cells, the overall RBE response is given by the blue curve.

A simulation of the skin results for protons is given below in **Figure 4**. This was obtained by scaling the proton RBE down to around 1.1 in the middle of the dose range and changing the radiosensitivity values to an RBEmax of around 1.3 and an RBEmin of just above unity as might be typical in the mid spread-out Bragg-Peak. The same conversion factor of 0.1 was applied to the excess of the RBE above 1 for each of the C3H and Ehrlich ascites carcinomas. **Figures 4**–**6** respectively show the simulations for normal skin, the C3H and Ehrlich carcinomas. In some instances, the modelling is extended beyond the data range to show the effect of each physiological compartment on the combined RBE. The assumed coefficient of variation for each example is the same as in the neutron simulations.

### 3.3 Repair rate and RBE

**Figure 7:**
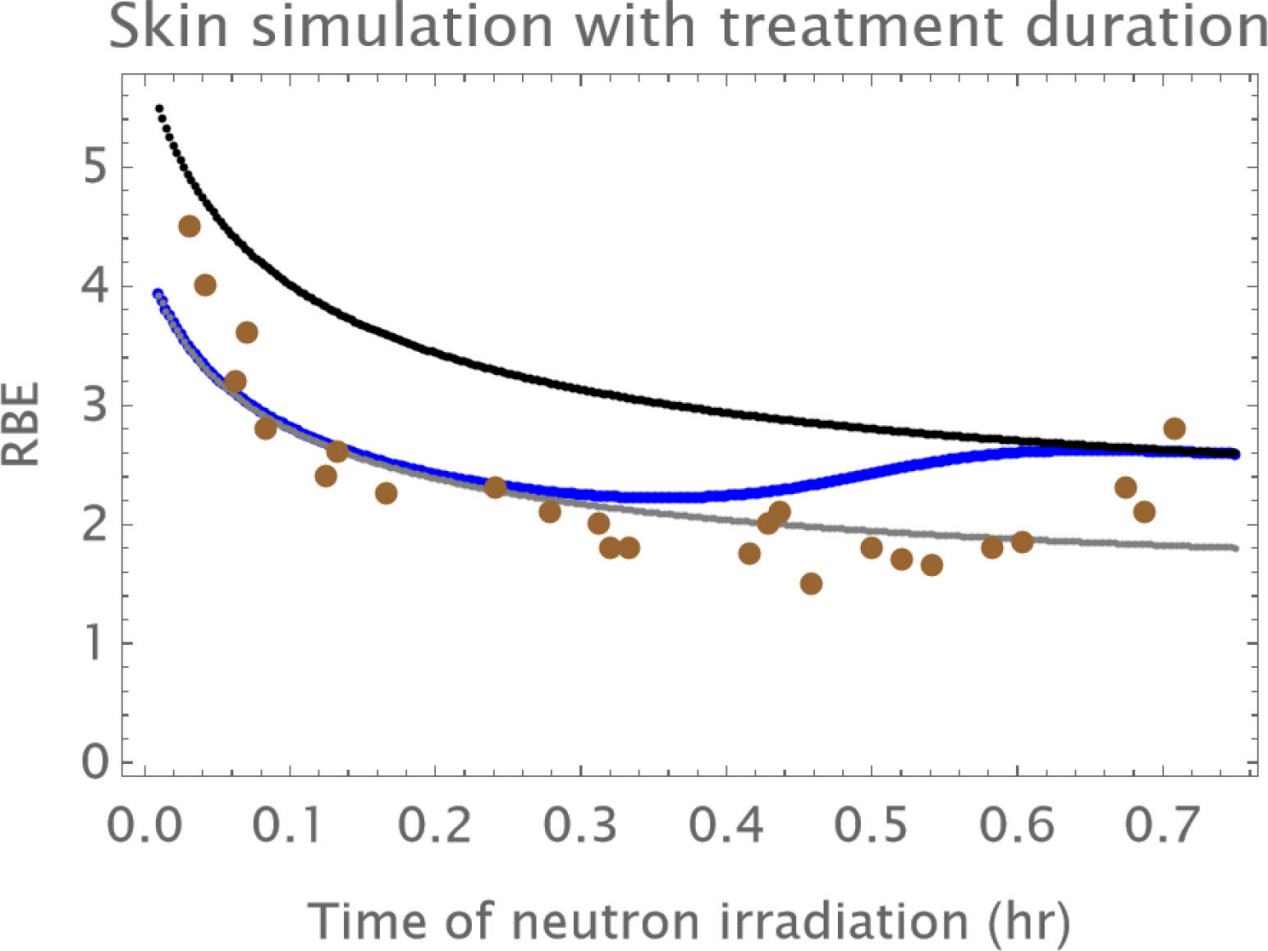
The treatment times for fast neutron therapy and the estimated RBE values for the Hammersmith skin data set are plotted with a DNA sub-lethal damage repair rate half-time of around 1 hour. This should be compared with **Figure 1**. Data points in brown; the grey curve is that of oxic cells, the black curve of hypoxic cells, the overall RBE response is given by the blue curve.

The influence of dose rate on RBE can be investigated similarly, as shown in **Figure 7**. With use of a time factor to act on the β parameter dose with inclusion of the oxic and hypoxic compartments for fast neutrons and the reference radiation, it is not possible to show a significant up-turn effect due to the dose rate effect over the duration of neutron treatment given at around 24 Gy/hour (as used in The Hammersmith experiments). This can be seen from the oxic only curve which does not appear to deviate upwards, so the presence of a hypoxic fraction appears to be necessary, although the RBEs are slightly raised with increasing dose if compared with **Figure 1**.

**Figure 8:**
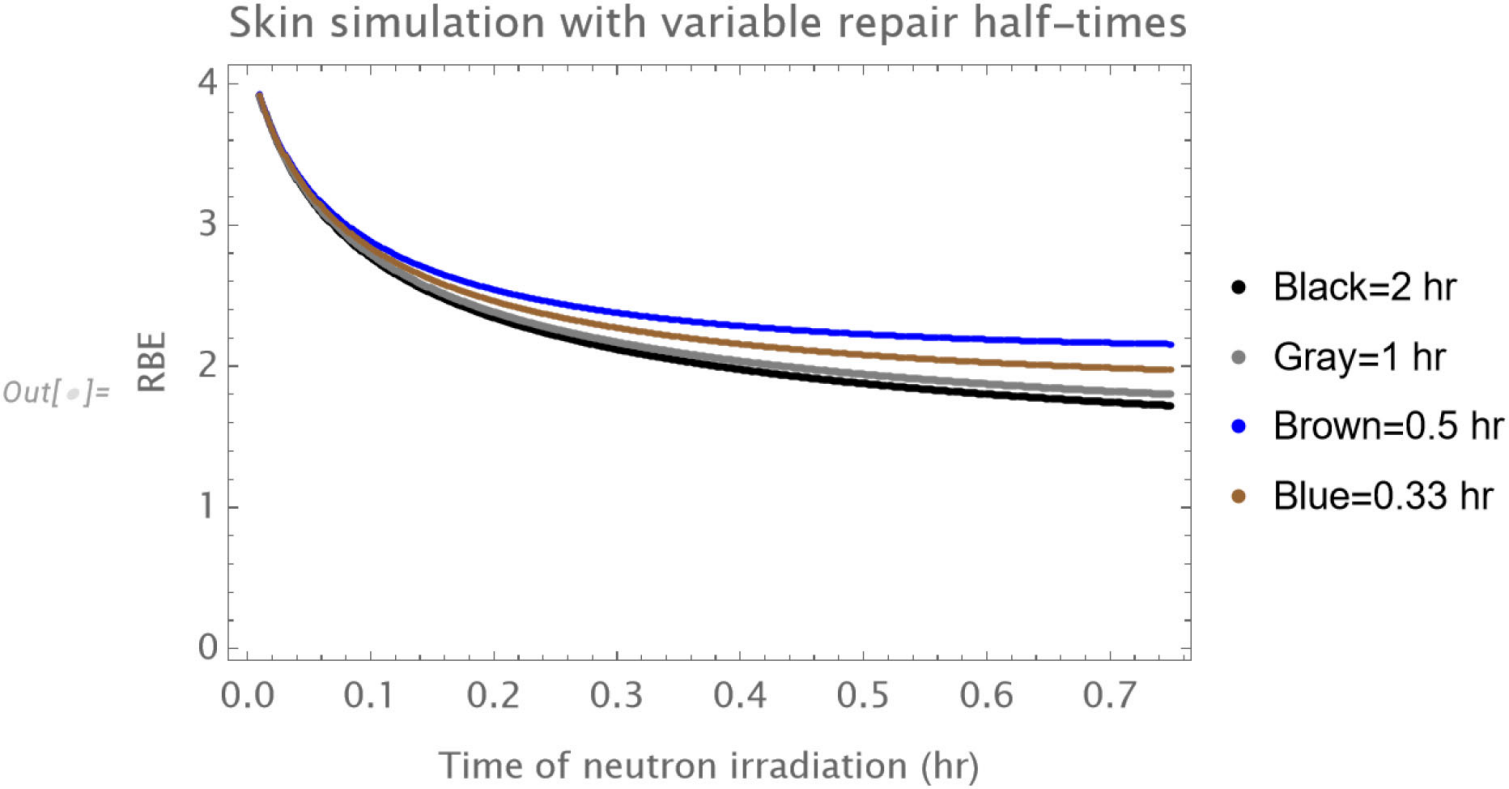
The influence of repair rate on RBE in an oxic cell population, with half times of repair shown as black (2hr), grey (1hr), brown (0.5 hour) and blue (0.33 hour). There is no upturn of RBE at longer durations, although RBE.

The theoretical influence of repair rate on RBE in purely oxic cells can be seen in **Figure 8**, where the half time of repair is varied between 20 mins, 0.5 hour, 1 hour and 2 hr. The RBE changes with treatment duration are all inversely related, but the magnitude of RBE is inversely related to the repair rate. Thus, it is concluded that the overall treatment time probably does not account for the substantial upturn in RBE seen in the Hammersmith skin experiments.

### 3.4 Other considerations

**Figure 9:**
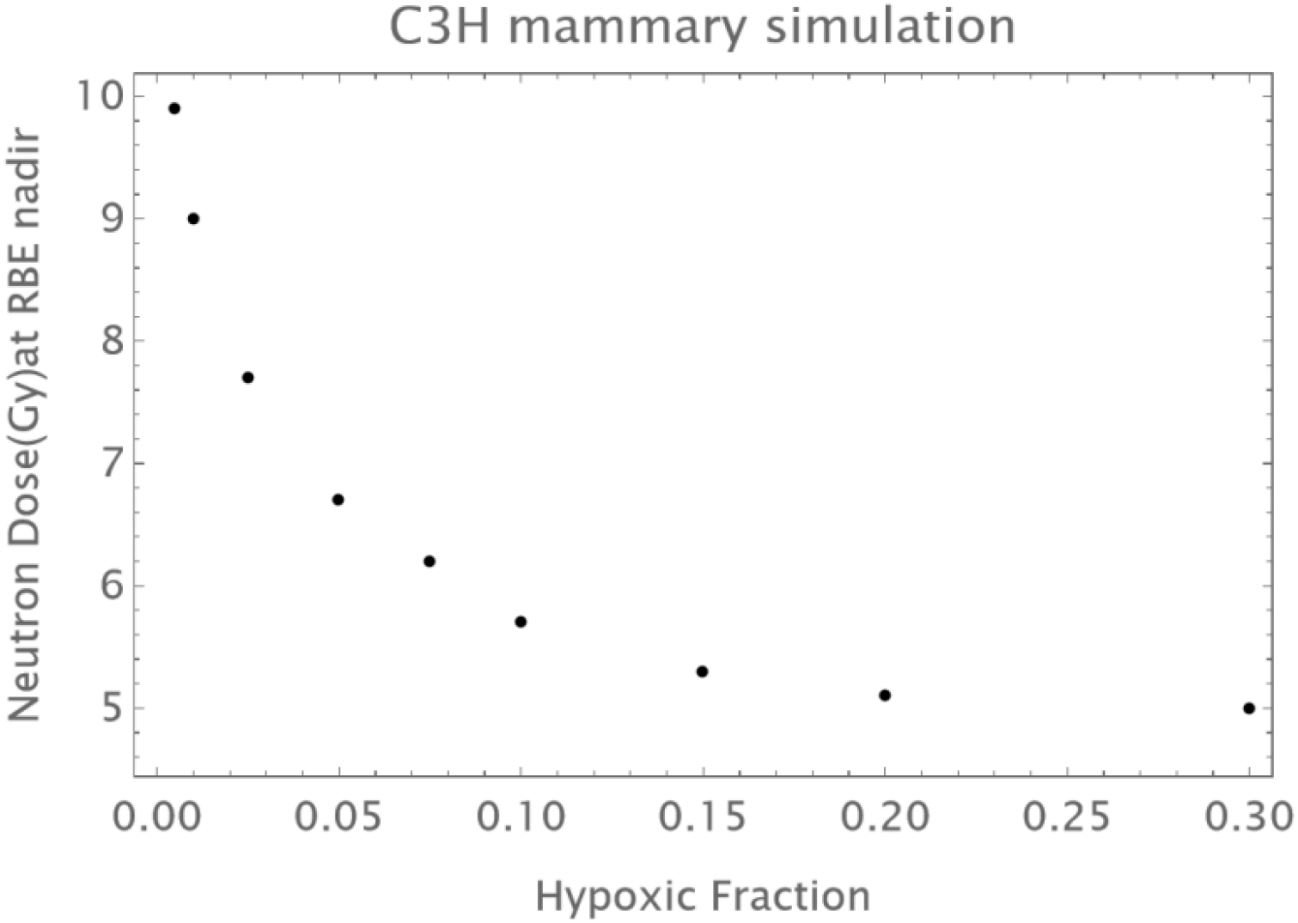
Example of a plot obtained by varying the hypoxic fraction, whilst maintaining constant values for all the other radiobiological parameters for the C3H carcinoma example, to obtain the minimum (nadir) RBE dose.

Analytical attempts using differential calculus to determine the dose (d_min_) at which the minimum value of RBE occurs is fraught with difficulty, due to the complexity of the final RBE equations. The main determining factor appears to be the hypoxic fraction (*h*), being inversely related to the minimum RBE. For example, if the other radiobiological parameters are constant, as would be the case for an individual tumor, the relationship between *h* and approximated d_min_ values is shown in **Figure 9** for the C3H carcinoma. The relationship can be fitted by the Equation 10. $e^{-4.04\;h}$ (*p* = 0.0003, *R^2^* = 0.97). The nadir RBEs are found by inspection of separate plots and since the nadir`s are broad will have some inherent inaccuracy. Variation of the oxygen related parameters can cause further subtle changes to these nadir points but to a lesser extent and are not shown here.

Plots of the relative numbers of surviving cells in each physiological compartment with increasing dose do not show any consistent relationship with d_min_ and are not reproduced here. Also, d_min_ does not occur when the combined RBE is at a central position between the oxic RBE and the hypoxic RBE. It appears that the shift from oxic to hypoxic curves is mainly determined by the hypoxic fraction but also modified by the higher LET values of *q* according to the experimental physiological conditions. The finding that the graphical outputs for protons can closely resemble the neutron results probably indicate that it is the relative ratios of the radiosensitivity and oxygen related parameters that govern what is a smaller shift in proton RBEs than occurs with neutron RBEs. Although cell killing is greater with neutrons than protons in the simulations the rate of change of RBE from oxic to hypoxic dominance appears to be similar.

## Discussion

The above modelled results show that, in three quite different biosystems, that the fast neutron RBE initially reduces with dose per fraction and can depend, at higher doses, on the hypoxic fraction and other oxygen related parameters. Protons may also show similar patterns, although the findings are highly speculative but sufficiently interesting and potentially important that further experimental work is indicated. The study provides insights into possible *in vivo* q_αH_ and q_βH_ values, although further research is required to provide more precise and direct information.

It would be of potential clinical importance to know if such changes occur in humans since hypofractionation could result in advantages where RBE has increased in a tumor at a dose where the RBE has continued to decrease in the normal tissue. Also, the use of very large fractions may not be optimal in the dose range of an elevated RBE in some normal tissues. At the present time proton therapy uses small dose fractions of around 2 Gy. Acute skin reactions around the eyelids, for example, can be problematic for proton treatments of uveal melanomas when the prescribed dose per fraction is often in the 11–14 Gy range and where the skin dose is influenced by the relative lack of skin sparing at the proximal spread-out Bragg peak due to the relatively low proton energy required to treat ocular targets usually within a 3-3.5 cm range (27). Such a dose range is just below the nadir for the mouse skin data, and at the present time it is not known if such a nadir occurs in humans. Laboratory proton skin experiments would seem to be justified to investigate this issue further.

Extrapolations from animal experiments to humans is difficult but where ratios such as RBE and OER determine outcomes there should be smaller differences, since ratios of these parameters are better preserved in different biosystems.

The dose rate effect should not be ignored in RBE estimations if treatments become prolonged due to their complexity and accelerator outputs (synchrotrons give lower dose rates than cyclotrons). Treatment duration can influence RBE although the inverse relationship between RBE and dose is maintained.

Other possible mechanisms for the RBE changes with dose must be considered. Dynamic changes in re-oxygenation would, for example, occur at a much later time and so cannot influence later RBE. However, late vascular effects might contribute to the tumor related changes. The tumor vasculature will also be exposed to temporal change in oxygenation and so be susceptible to increased RBE at higher doses, just as the tumor cells are. The higher late effect RBE in the vascular epithelium which would manifest more at higher doses (and be more pronounced with neutrons) could presumably cause enhanced tumor growth delay, elevating RBE, but would depend on the onset time of tumor vascular damage. Such a possibility could be tested by experiment with detailed vascular status monitoring and histological studies. However, the fact that the Ehrich ascites cancer does show the influence of a large initial hypoxic fraction and cannot be directly influenced by vascular effects strongly suggests that such a mechanism is not responsible for increases in RBE with dose per fraction in rapidly growing tumors. It must be recalled that the onset time of classical late effects occurs at least several months after exposure, even in experimental animals, and the recurrence times for fast growing experimental cancers would occur at shorter times.

The issue of LET spatial heterogeneity is of current interest but is perhaps beyond the scope of the present article although, in principle, this could be addressed by a detailed modelling study in proton experiments. The Hammersmith neutrons had a similar dose distribution to 300 KeV x-rays, so that the fall-off of dose would have been small across a 1–2 cm tumor or through skin, with little or no change in LET. Proton experiments would require relatively low energies for the tumor experiments but higher energies for an ascites cancer; they would probably be simple single field set-ups in experiments, such that the LET distribution would be known. RBEs would change in the sub-volumes of interest by amounts directly related to the LET but also modified by the oxygen-status of the sub-volume. To take this further would require detailed mapping of LET, dose and of the oxic and hypoxic zones during experimental irradiations, and where changes in LET distribution can be accommodated.

FLASH (ultra-high dose rate) effects should also be considered. Any predictions would be highly speculative. FLASH effects do require a high dose per fraction, and much would depend on if electron FLASH is compared with Proton FLASH, or conventional dose-rate Megavoltage photons with proton FLASH. Depletion of local oxygen in tumor oxic zones by FLASH irradiation (28) might confer the higher RBE of the hypoxic zone to prevail throughout a tumor (or within a normal tissue, whilst simultaneously changing normal tissue radiotolerances). This is a complex field where considerable experimental work and in-silico modelling would be necessary.

The new overall model is radiobiologically complex (more than 16 equations and other identities are required to describe it adequately). It will be regarded by some experts as heuristic, although its theoretical basis is considerable and based on a realistic science base given by extant knowledge of classical radiobiology. The weakness lies in the possible lack of at least some of the key representative parameter precision, such that the quantitative information gained above has been reached by a tentative approach, although handled with great care and by observing realistic constraints. However, it has been necessary in many branches of the physical sciences to make advances by theoretical modelling and tentative approaches where complexity is considerable. Such work can help to clarify the design of future experiments and the key parameters which need to be studied further. The data being fitted has many limitations, such as the way in which it was published as graphics without data points and future experiments could overcome this retrospective problem.

From the above discussion, there are sufficient reasons for pursuing a large portfolio of further experimental research, as already outlined.

## Data Availability

The raw data supporting the conclusions of this article will be made available by the authors, without undue reservation.

## Appendix

The oxic d_L_ is $\frac{\alpha_{L}+\sqrt{\alpha_{L}^{2}+4\alpha_{H\;\;}\beta_{L\;}d_{H}+4\beta_{H}\;\beta_{L}\;d_{H}^{2}}}{2\beta_{L}}$The hypoxic d_L_ is $\frac{0.5\;(-\;\frac{\alpha_{L}\;q_{\beta }}{q_{\alpha }}+\sqrt{\frac{q_{\beta }^{2}\;\alpha_{L\;}^{2}}{q_{\alpha }^{2}}-4\beta_{L}\;q_{\beta }\;G}}{\beta_{L}}$,

Where G is the natural logarithm of $\frac{0.8d_{H}}{q_{\alpha n}}-\frac{0.05}{q_{bn}}d_{H}^{2}$The RBEs will be the respective d_L_ values divided by d_H_ in each case.
